# Impact of transversus abdominis plane block plus intravenous lidocaine on rapid recovery after bariatric surgery

**DOI:** 10.3389/fmed.2025.1694309

**Published:** 2025-11-26

**Authors:** Xin Wang, Yawen Zhang, Qiongmei Guo, Li Wang, Jin Zhang, Zhenheng Yang, Yuan Sun

**Affiliations:** Department of Anesthesiology, The First Hospital of Hebei Medical University, Shijiazhuang, Hebei, China

**Keywords:** bariatric surgery, transversus abdominis plane block, lidocaine, analgesia, rapid recovery

## Abstract

**Aim:**

The study aimed to explore the impact of transversus abdominis plane block (TAPB) combined with intravenous lidocaine on rapid recovery after bariatric surgery.

**Methods:**

A total of 80 patients who underwent elective bariatric surgery from October 2022 to October 2023 were selected as study participants and divided into a control group (CG) and a study group (SG). The CG received ultrasound-guided TAPB with ropivacaine, while the SG received ultrasound-guided TAPB with ropivacaine plus intravenous lidocaine. The two groups were compared in terms of total doses of propofol, remifentanil, and sufentanil used; pain intensity; intraoperative conditions; levels of inflammatory factors; additional use of postoperative analgesics; incidence of adverse reactions; and postoperative recovery time.

**Results:**

Compared to the CG, the SG showed significant reductions in the total intraoperative doses of propofol, remifentanil, and sufentanil (*p* < 0.05). In addition, the SG had lower visual analog scale (VAS) scores at rest and during coughing at 12, 24, and 48 h postoperatively (*p* < 0.05), as well as lower heart rate (HR) and mean arterial pressure (MAP) values at time points T1–T3 (*p* < 0.05). On the first day after surgery, the levels of tumor necrosis factor-α (TNF-α) and interleukin 6 (IL-6) in the SG were significantly lower than those in the CG (*p* < 0.05), and the SG also required less additional use of postoperative analgesics (*p* < 0.05). Furthermore, the SG exhibited a shorter time to first flatus, first defecation, and first ambulation, as well as a shorter length of hospital stay compared to the CG (all *p* < 0.05).

**Conclusion:**

TAPB combined with intravenous lidocaine may provide effective postoperative analgesia for patients after bariatric surgery and may accelerate their rapid recovery.

## Introduction

With the evolution of social development and lifestyle changes, obesity has become one of the most serious epidemics of the 21st century (1). According to the Report on Nutrition and Chronic Diseases of Chinese Residents (2020), the prevalence of overweight and obesity among adult residents in China exceeds 50%, while the rate among children and adolescents aged 6–17 years is close to 20%. Both the incidence and growth rate of obesity in China rank first globally (2). Obesity not only increases cardiac burden, reduces lung compliance, impairs gastrointestinal motility, and elevates the risk of kidney damage but also leads to a series of complications, such as sleep apnea syndrome, type 2 diabetes mellitus, hyperlipidemia, hypertension, and hyperuricemia (3). Obesity not only increases cardiac burden, reduces lung compliance, impairs gastrointestinal motility, and elevates the risk of kidney damage but also leads to a series of complications, such as sleep apnea syndrome, type 2 diabetes mellitus, hyperlipidemia, hypertension, and hyperuricemia (4). Patients undergoing bariatric surgery are prone to postoperative complications, including nausea, vomiting, respiratory depression, delayed gastrointestinal function recovery, and anastomotic fistula (5).

At present, the primary regional block technique for patients undergoing bariatric surgery is transversus abdominis plane block (TAPB), which can reduce perioperative opioid consumption, alleviate postoperative pain, and improve patient comfort (6). Recent studies have shown that perioperative intravenous administration of lidocaine offers certain benefits in relieving pain and reducing postoperative nausea and vomiting (PONV) (7). In addition, there is evidence that intravenous lidocaine can promote the recovery of postoperative gastrointestinal function in patients undergoing gastrointestinal surgery (8). However, due to excessive fat accumulation and associated pathophysiological changes in obese patients, the impact of intravenous lidocaine infusion on bariatric surgery outcomes remains controversial (9).

Therefore, our study aimed to explore the effect of TAPB combined with intravenous lidocaine on the rapid recovery of patients after bariatric surgery.

## Data and methods

### General data

A total of 80 patients who underwent elective bariatric surgery from October 2022 to October 2023 were selected as study participants. The inclusion criteria were as follows: (1) Age >18 years, (2) American Society of Anesthesiologists (ASA) physical status classification II to III, and (3) body mass index (BMI) > 30 kg/m^2^. All patients were transferred to the post-anesthesia care unit (PACU) after surgery and received patient-controlled intravenous analgesia (PCIA). The exclusion criteria were as follows: (1) Contraindications to nerve block, (2) allergy to local anesthetics, (3) severe organic diseases, (4) chronic use of opioid analgesics, (5) conversion to open surgery during the operation, (6) mental illness or communication disorders, and (7) operation duration >3 h. The withdrawal criteria included patients who voluntarily withdrew from the study or experienced adverse events, such as massive intraoperative bleeding.

The patients were randomly allocated to the control group (CG) and the study group (SG) using a random number table, with 40 cases in each group. The CG included 15 male and 25 female individuals, aged 20–42 years, with a mean age of (31.83 ± 6.72) years. Their BMI ranged from 31 to 60 kg/m^2^, with a mean BMI of (42.12 ± 6.15) kg/m^2^. The SG included 16 male and 24 female individuals, aged 21–43 years, with a mean age of (31.78 ± 6.70) years. Their BMI ranged from 31 to 62 kg/m^2^, with a mean BMI of (42.15 ± 6.18) kg/m^2^. No statistically significant difference was observed in general data between the two groups (*p* > 0.05).

### Method of anesthesia

Electrocardiogram (ECG), blood oxygen saturation (SpO₂), non-invasive blood pressure, and bispectral index (BIS) were monitored, and oxygen was administered to all patients. Peripheral venous access was established in the upper limb, followed by sedation with midazolam and sufentanil. Invasive arterial pressure monitoring was performed via radial artery puncture under local anesthesia, and arterial blood samples were collected for blood gas analysis. During anesthesia induction, all patients in both groups received midazolam (0.05 mg/kg), sufentanil (0.3–0.5 μg/kg, based on ideal body weight), etomidate (0.15–0.3 mg/kg, based on actual body weight), and rocuronium bromide (1 mg/kg, based on ideal body weight). After tracheal intubation, a temperature probe was inserted through the nasal cavity to continuously monitor nasopharyngeal temperature. For anesthesia maintenance, the patients received continuous intravenous infusions of propofol [4–8 mg/(kg·h), based on actual body weight] and remifentanil [0.1–0.3 μg/(kg·min), based on ideal body weight] to maintain a BIS value of 40–60 and a surgical pleth index (SPI) of 25–50. Intermittent intravenous injections of rocuronium bromide (0.2 mg/kg) were also administered. Vasoactive drugs were adjusted according to circulatory status to maintain blood pressure and heart rate (HR) within ±20% of baseline values. Before the end of the operation, all patients received nalbuphine hydrochloride (5 mg) and azasetron hydrochloride (10 mg). After surgery, an analgesic pump was connected, and the patients were transferred to the post-anesthesia care unit.

The CG received ultrasound-guided TAPB with 40 mL of 0.3% ropivacaine. In addition, 1.5 mg/kg of normal saline was administered intravenously as a bolus, and a continuous intravenous infusion of normal saline (1.5 mg/kg/h) was maintained throughout the operation.

The SG also received ultrasound-guided TAPB with 40 mL of 0.3% ropivacaine. In addition, 1.5 mg/kg of lidocaine was administered intravenously as a bolus, and a continuous intravenous infusion of lidocaine (1.5 mg/kg/h) was maintained throughout the operation.

### Observation indicators

The total intraoperative doses of propofol, remifentanil, and sufentanil were recorded and compared between the two groups. The visual analog scale (VAS) was used to evaluate the degree of pain under two distinct conditions: (1) pain at rest (spontaneous pain while stationary) and (2) pain on coughing (incident pain triggered by a forced, voluntary cough). Assessments were performed at 3 h, 6 h, 12 h, 24 h, and 48 h after surgery.

Mean arterial pressure (MAP), heart rate (HR), blood oxygen saturation (SpO₂), and bispectral index (BIS) values were recorded for both groups at four time points: 15 min before surgery (T0), immediately after pneumoperitoneum establishment (T1), immediately after gastrectomy completion (T2), and at extubation (T3). The levels of inflammatory factors, including tumor necrosis factor-α (TNF-α) and interleukin 6 (IL-6), were measured using enzyme-linked immunosorbent assay (ELISA) with commercial kits (TNF-α Kit: Nanjing Jiancheng Bioengineering Institute, Nanjing, China, Catalog No. H052-1-2; IL-6 Kit: Beyotime Biotechnology, Shanghai, China, Catalog No. PI326). The additional use of postoperative analgesics was compared between the two groups. The incidence of adverse reactions, including nausea, vomiting, respiratory depression, hypoxemia, dizziness, and hypotension, was recorded in both groups. Postoperative recovery indicators, including time to first flatus, time to first defecation, time to first ambulation, and length of hospital stay, were recorded and compared between the two groups.

### Statistical analysis

Statistical analysis was performed using the SPSS 24.0 software. The normality of the distribution for measurement data was assessed using the Shapiro–Wilk test, and the homogeneity of variances was verified using Levene’s test. Measurement data were expressed as mean ± standard deviation (x̄ ± s). Comparisons between the two groups at the same time points were conducted using an independent samples *t*-test. Comparisons within the same group before and after surgery were performed using a paired samples *t*-test. Categorical data were presented as counts and percentages (*n*, %), and group comparisons were analyzed using the chi-squared (*χ*^2^) test. A *p*-value of <0.05 was considered to indicate a statistically significant difference.

## Results

### Total doses of propofol, remifentanil, and sufentanil administered during the operation in the two groups

**Figure 1:**
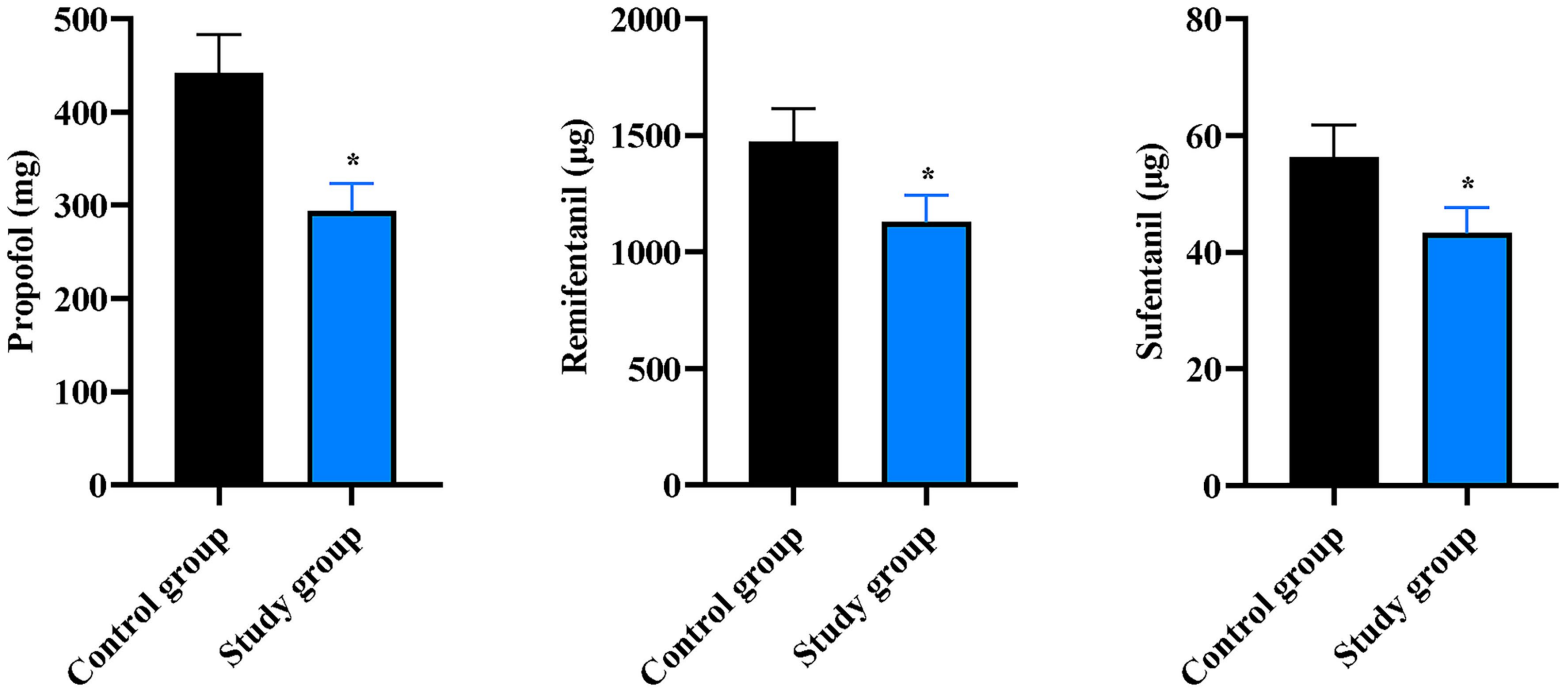
Total doses of propofol, remifentanil, and sufentanil administered during the operation between the two groups. Data are presented as mean ± standard deviation; *n* = 40 per group; ^*^p < 0.05.

The consumption of key anesthetic agents during surgery was significantly lower in the SG compared to the CG. The total dose of propofol in the CG was 441.85 ± 41.36 mg, which was significantly higher than the 345.60 ± 52.10 mg observed in the SG (*p* < 0.05). Similarly, the total dose of remifentanil in the SG was markedly lower than that in the CG (1129.32 ± 112.35 μg vs. 1472.00 ± 142.65 μg, *p* < 0.05). Furthermore, the total dose of sufentanil administered was also lower in the SG compared to the CG (43.28 ± 4.32 μg vs. 56.21 ± 5.62 μg, *p* < 0.05) (Figure 1).

### VAS scores between the two groups

**Figure 2:**
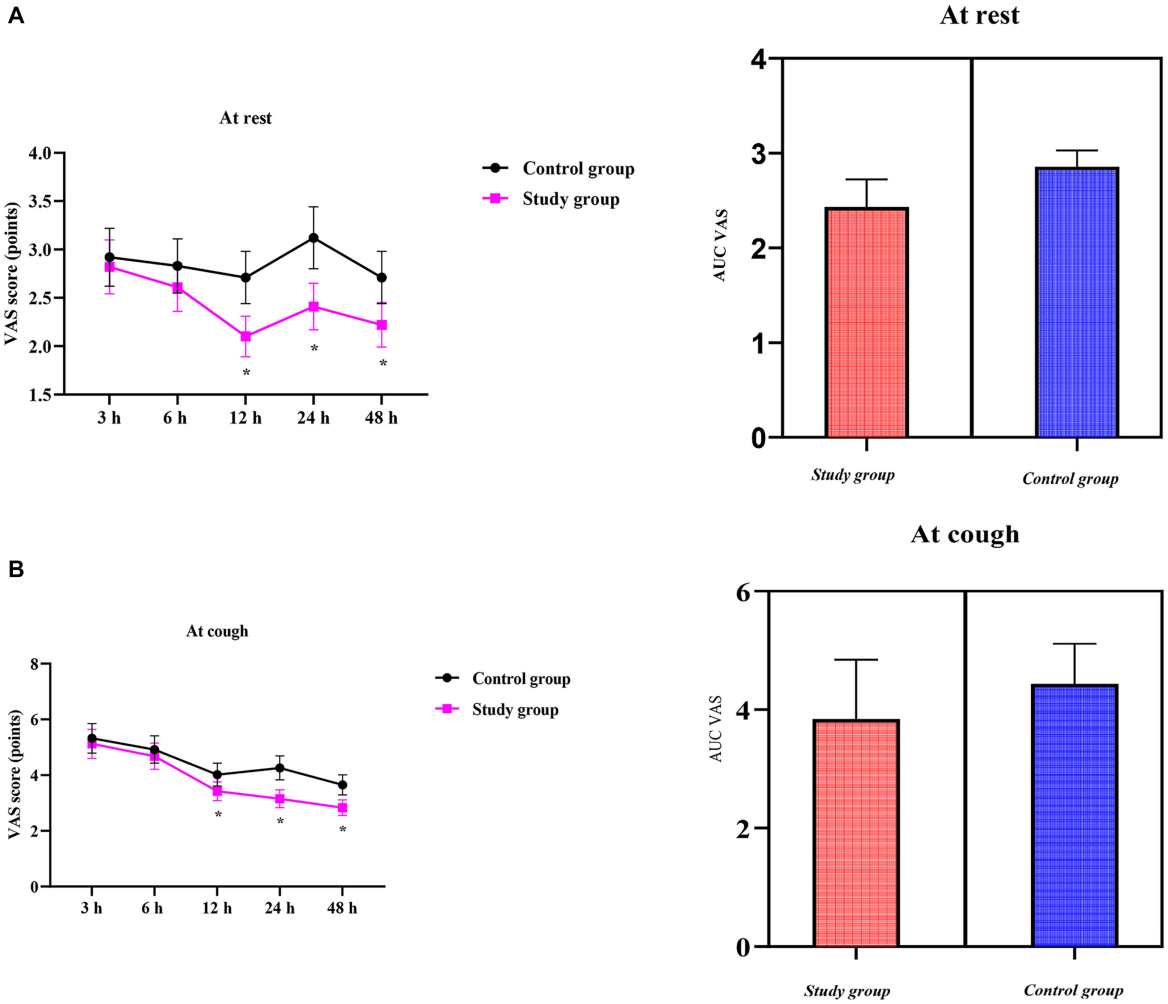
Visual analog scale (VAS) scores over time and the area under the curve (AUC) of VAS scores between the two groups; **(A)** at rest; **(B)** on coughing. Data are presented as mean ± standard deviation; *n* = 40 per group; ^*^p < 0.05.

VAS scores at rest were similar between the two groups in the early postoperative period (3 h: CG 2.92 ± 0.30 vs. SG 2.82 ± 0.28, *p* > 0.05; 6 h: 2.83 ± 0.28 vs. 2.61 ± 0.25, *p* > 0.05). Thereafter, the SG reported significantly less pain at rest, with differences reaching both statistical and clinical significance at 12 h (2.71 ± 0.27 vs. 2.10 ± 0.21, *p* < 0.05), 24 h (3.12 ± 0.32 vs. 2.41 ± 0.24, *p* < 0.05), and 48 h (2.71 ± 0.27 vs. 2.22 ± 0.23, *p* < 0.05) (Figure 2).

Compared to the CG, the SG showed consistently lower VAS scores regarding coughing throughout the observation period. Pain on coughing was comparable between the two groups during the early phase (3 h: CG 5.32 ± 0.53 vs. SG 5.12 ± 0.52; 6 h: 4.92 ± 0.49 vs. 4.68 ± 0.47, both *p* > 0.05). Thereafter, the SG reported significantly milder cough-related pain, with differences reaching both statistical and clinical significance at 12 h (4.02 ± 0.41 vs. 3.42 ± 0.34), 24 h (4.26 ± 0.43 vs. 3.15 ± 0.32), and 48 h (3.65 ± 0.36 vs. 2.83 ± 0.28) (all *p* < 0.05) (Figure 2).

### Intraoperative condition of the two groups

No differences were observed in HR, MAP, SpO_2,_ and BIS at T_0_ between the two groups (*p* > 0.05).

**Figure 3:**
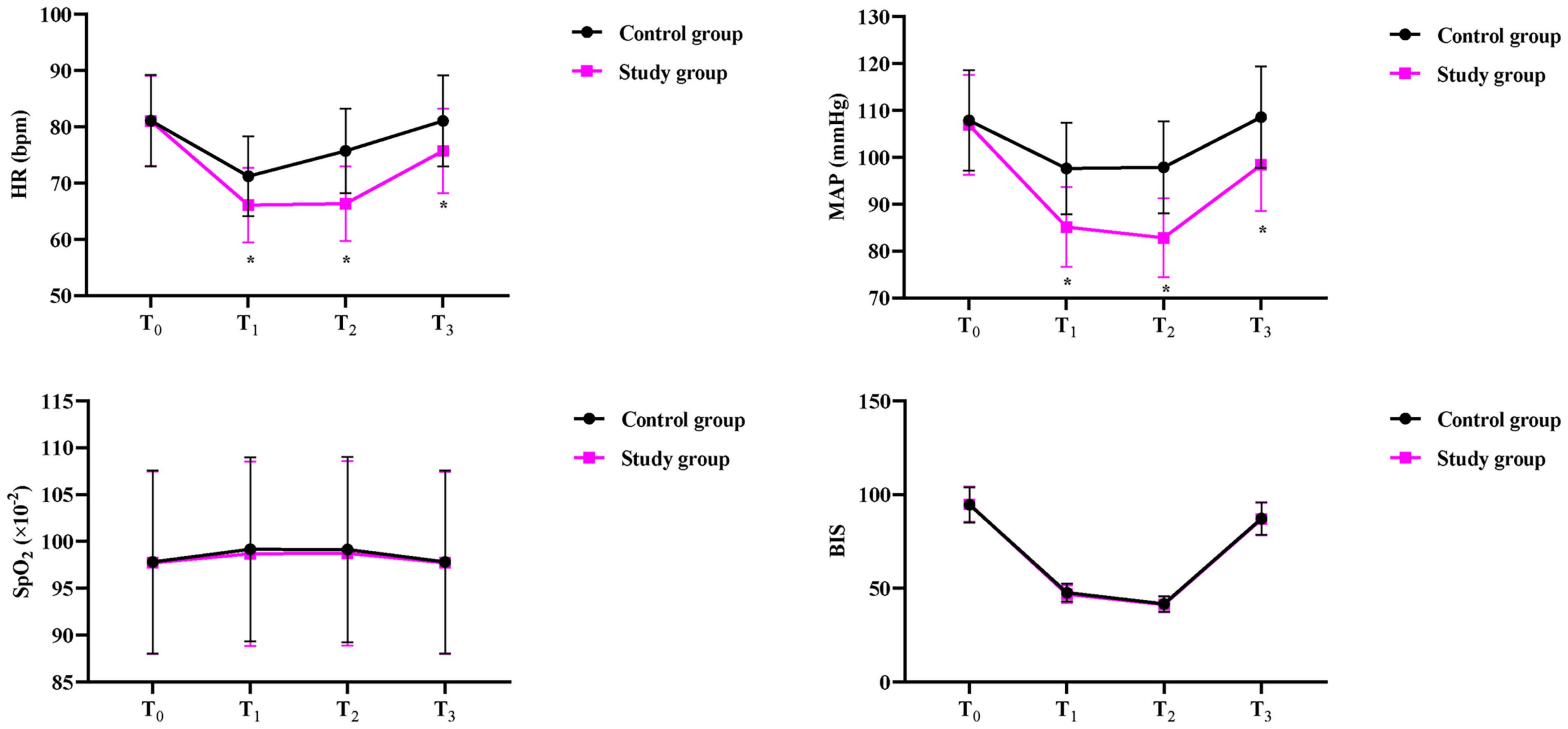
Intraoperative hemodynamic parameters between the two groups. Heart rate (HR) and mean arterial pressure (MAP) were recorded at different time points: 15 min before surgery (T₀), after pneumoperitoneum completion (T₁), after gastrectomy completion (T₂), and at extubation (T₃). SpO_2_: blood oxygen saturation, BIS: bispectral index. Data are presented as mean ± standard deviation (*n* = 40 per group); ^*^p < 0.05.

Compared to the CG, the SG showed significantly lower HR values at T₁–T₃ (T₁: 66.10 ± 6.62 vs. 71.23 ± 7.12 bpm; T₂: 66.35 ± 6.62 vs. 75.73 ± 7.52 bpm; T₃: 75.73 ± 7.54 vs. 81.05 ± 8.13 bpm; all *p* < 0.05) (Figure 3). Throughout surgery (T₁–T₃), the SG maintained consistently lower blood pressure than the CG: (T₁: 85.12 ± 8.52 vs. 97.62 ± 9.75 mmHg; T₂: 82.85 ± 8.36 vs. 97.86 ± 9.82 mmHg; T₃: 98.36 ± 9.83 vs. 108.56 ± 10.82 mmHg; all *p* < 0.05) (Figure 3).

### Inflammatory response between the two groups

**Figure 4:**
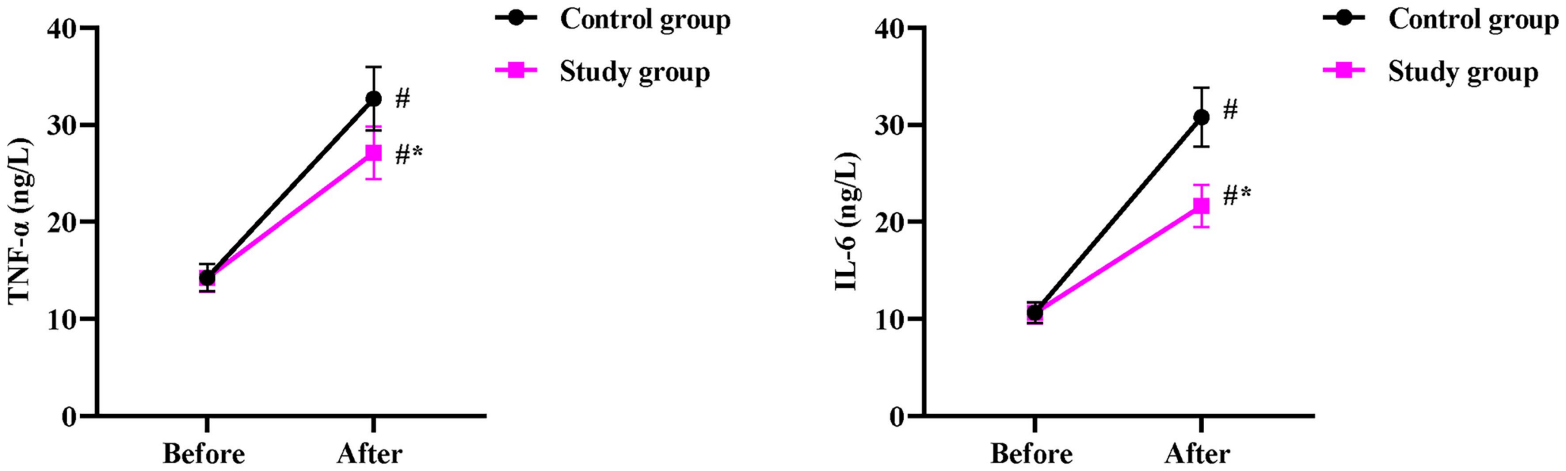
Postoperative inflammatory response between the two groups. Serum levels of TNF-α and IL-6 were measured before and 1 day after surgery. Data are presented as mean ± standard deviation (*n* = 40 per group). Intra-group comparisons (before vs. after surgery) were analyzed using a paired samples *t*-test, and inter-group comparisons at the same time point were analyzed using an independent samples *t*-test; ^*^p < 0.05.

Before surgery, no significant differences were observed in serum TNF-α and IL-6 levels between the two groups (*p* > 0.05). One day after surgery, both groups showed a marked rise in TNF-α (*p* < 0.05), but the SG exhibited a significantly smaller increase (27.12 ± 2.71 ng/L) than the CG (32.68 ± 3.26 ng/L, *p* < 0.05, Figure 4). Similarly, one day after surgery, IL-6 concentrations increased significantly in both groups (*p* < 0.05), but the elevation was markedly attenuated in the SG (21.62 ± 2.16 ng/L) compared to the CG (30.78 ± 3.08 ng/L, *p* < 0.05, Figure 4).

### Additional use of postoperative analgesics between the two groups

Compared to the CG, the SG had a significantly lower rate of additional postoperative analgesic use (*p* < 0.05, Table 1).

**Table 1:** Additional use of postoperative analgesics in the two groups.

Group	No. of patients (*N*)	Patients requiring rescue analgesics *n* (%)
Control group	40	20 (50.0)
Study group	40	8 (20.0)
*χ* ^2^		7.912
*p-*value		0.004

Data are presented as the number of patients (*n*) and percentage (%). Group comparisons were performed using the chi-squared test.

### Incidence of adverse reactions between the two groups

No significant difference was discovered in the incidence of adverse reactions between the two groups (*p* > 0.05, Table 2).

**Table 2:** Incidence of adverse reactions in the two groups.

Adverse reaction	Control group (*N* = 40)	Study group (*N* = 40)	*χ* ^2^	*p*-value
Nausea and vomiting	1	2		
Respiratory depression	1	1		
Hypoxemia	1	1		
Dizziness	1	1		
Hypotension	1	1		
Total incidence	5 (12.50%)	6 (15.00%)	0.105	0.745

Adverse reactions were recorded during the first 24 h postoperatively. Data are presented as the number of patients. The total incidence was compared between the groups using the chi-squared test.

### Postoperative recovery time in the two groups

**Figure 5:**
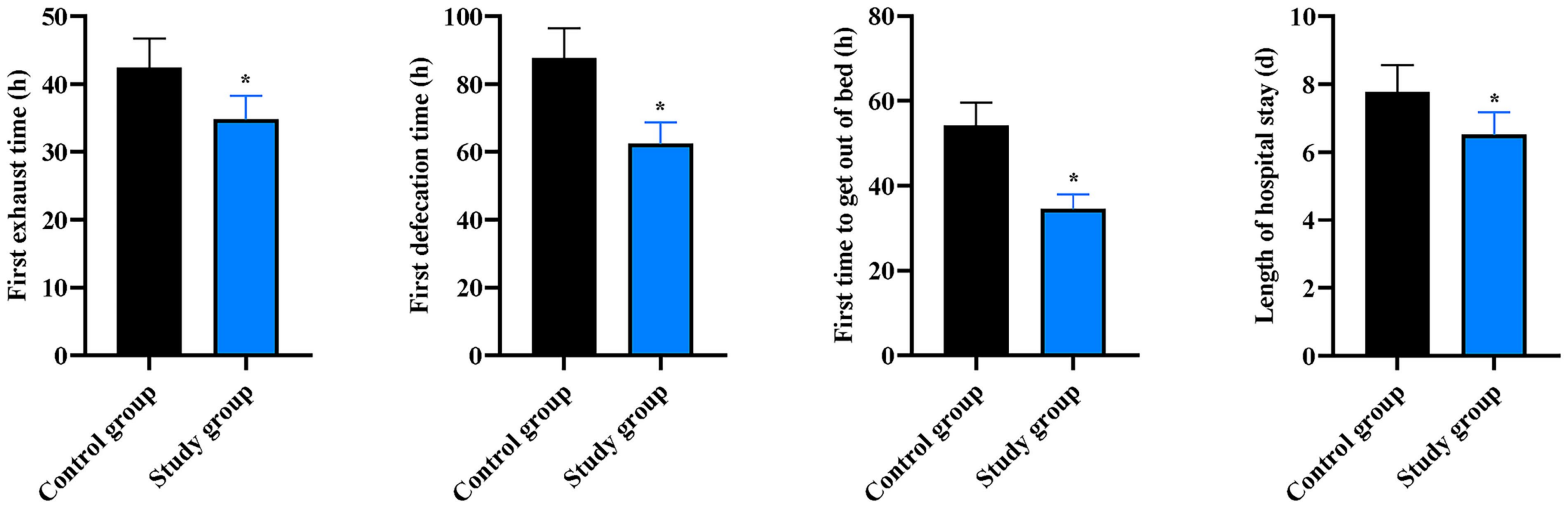
Postoperative recovery outcomes between the two groups. Comparison of first flatus time, first defecation time, time to first ambulation, and length of hospital stay between the two groups. Data are presented as mean ± standard deviation (*n* = 40 per group). *p < 0.05 vs. Control group.

Compared to the CG, the SG had significantly shorter times for first flatus (42.45 ± 4.26 h vs. 34.82 ± 3.48 h), first defecation (87.65 ± 8.78 h vs. 62.43 ± 6.25 h), first ambulation (54.23 ± 5.42 h vs. 34.52 ± 3.45 h), and length of hospital stay (7.78 ± 0.78 day vs. 6.52 ± 0.65 day) (*p* < 0.05, Figure 5).

## Discussion

In recent years, overweight and obesity have become global public health problems. According to reports, the prevalence of overweight and obesity among Chinese adult residents is 34.3 and 16.4%, respectively, and the overall overweight and obesity rate in China is currently showing a rapid upward trend (10). At present, bariatric and metabolic surgery is more precise and effective than medical therapy for the management of morbid obesity and its associated complications (11). Currently, the main bariatric and metabolic surgical procedures primarily include laparoscopic sleeve gastrectomy, laparoscopic Roux-en-Y gastric bypass, and biliopancreatic diversion/duodenal switch (12). Compared to patients with normal body weight, obese patients have excessive fat accumulation and associated pathophysiological changes, which can alter the pharmacokinetics of anesthetic drugs, increase required drug doses, and elevate the risk of adverse reactions such as drug accumulation, nausea, vomiting, and respiratory depression—all of which are unfavorable for the postoperative rapid recovery of obese patients (13) The most common complication is postoperative nausea and vomiting (PONV), with an incidence of approximately 65%, which is significantly higher than that in normal-weight adults (14). PONV may affect patients’ postoperative eating and can even lead to wound dehiscence, incisional hernia, aspiration pneumonia, and water and electrolyte imbalances, all of which can prolong hospital stay, increase medical costs, and lead to decreased patient satisfaction (15). In addition, obese patients have higher body weight and increased fat mass, which affect the distribution, protein binding, and excretion of anesthetic drugs. As a result, obese patients require higher doses of opioids, and opioids themselves further increase the incidence of PONV (16). Recently, a large body of literature has indicated that opioids inherently have the side effect of inhibiting gastrointestinal peristalsis, and opioid-induced intestinal dysfunction can lead to postoperative nausea and vomiting—an outcome that is detrimental to the recovery of gastrointestinal function (17).

The accumulation of fat in the chest and abdomen of obese patients obscures anatomical landmarks, making the performance of nerve blocks and neuraxial anesthesia more challenging. Currently, general anesthesia is the primary anesthetic method used in bariatric surgery (18). With the continuous advancement of ultrasound technology, general anesthesia combined with nerve blocks has been gradually applied in patients undergoing bariatric procedures (19). Recent studies have demonstrated that TAPB can improve surgical conditions for bariatric surgery, shorten operation duration, reduce the dose of muscle relaxants, and significantly enhance the quality of postoperative recovery within 24 h after surgery. In addition, it alleviates early postoperative pain, decreases opioid consumption, improves postoperative sleep quality, and promotes overall postoperative recovery in patients (20). However, TAPB typically only blocks abdominal wall nerves and has a limited effect on visceral nerves (21).

Lidocaine, a commonly used local anesthetic, plays an irreplaceable role in analgesia, immune regulation, anti-inflammation, and anti-arrhythmic effects (22). Both domestic and international studies have confirmed that perioperative intravenous lidocaine offers certain benefits in alleviating pain and reducing postoperative nausea and vomiting (23). However, in patients undergoing bariatric surgery, due to excessive fat accumulation and associated pathophysiological changes, the effects of intravenous lidocaine infusion have been rarely reported. Moreover, the use of intravenous lidocaine combined with Enhanced Recovery After Surgery (ERAS) in bariatric surgery—both domestically and internationally—remains limited, with a lack of sufficient clinical evidence to support its efficacy.

In our study, the results showed that, compared to the CG, the SG had significantly lower total intraoperative doses of propofol, remifentanil, and sufentanil, as well as reduced VAS scores at rest and during coughing at 12, 24, and 48 h postoperatively. In addition, HR and MAP values at time points T1–T3 were lower in the SG. These findings suggest that TAPB combined with intravenous lidocaine can reduce the consumption of propofol, remifentanil, and sufentanil; alleviate pain; and stabilize HR and MAP in patients after bariatric surgery—consistent with the results of previous studies (24, 25).

The inflammatory response plays a critical role in postoperative recovery. Surgical trauma and postoperative pain can trigger the release of large quantities of inflammatory factors, which not only increase postoperative hyperalgesia and exacerbate pain but also cause immune suppression and impede the postoperative recovery process (26). TNF-α and IL-6 are typical pro-inflammatory factors, and their expression levels can reflect the degree of systemic inflammation (27). In our study, compared to the CG, the SG exhibited lower TNF-α and IL-6 levels on the first day after surgery, required less additional postoperative analgesics, and had shorter times to first flatus, first defecation, first ambulation, and length of hospital stay. Collectively, these results indicate that TAPB combined with intravenous lidocaine can mitigate the inflammatory response and promote postoperative recovery in patients after bariatric surgery.

Consistent with our findings, previous studies have reported that TAPB and lidocaine can inhibit the postoperative inflammatory response and facilitate early recovery (28, 29). Specifically, studies have suggested that opioids induce pain sensitization, which, in turn, increases the release of inflammatory factors. In the present study, TAPB and lidocaine reduced opioid consumption, thereby inhibiting the inflammatory response. Furthermore, delayed postoperative recovery of intestinal function may be triggered by trauma- or surgery-induced inflammatory responses; lidocaine can block this process by inhibiting neurotransmission in injured tissues and regulating the balance between pro-inflammatory and anti-inflammatory cytokines (30). Meanwhile, lidocaine exerts a direct excitatory effect on intestinal smooth muscle, which can block the inhibitory reflex of the myenteric plexus and shorten the duration of postoperative ileus (31).

Beyond the statistically significant improvements in analgesic and recovery parameters, our findings have considerable clinical relevance. The incorporation of intravenous lidocaine into a multimodal analgesic regimen centered on TAPB appears to be a simple and safe strategy that synergistically addresses several challenges in post-bariatric care. The marked reduction in perioperative opioid requirements is clinically paramount, as it directly translates into a lower risk of opioid-induced adverse effects, particularly postoperative nausea and vomiting, which is exceptionally prevalent and distressing in this patient population. Furthermore, the accelerated recovery of gastrointestinal function, evidenced by shorter times to first flatus and first defecation, represents a critical milestone. It not only enhances early patient comfort but may also allow for earlier initiation of oral nutrition, a key component of the ERAS protocols. This, combined with significantly shorter times to ambulation and reduced hospital stay, contributes to a more efficient postoperative trajectory, potentially decreasing healthcare costs and increasing patient satisfaction. The attenuated systemic inflammatory response observed may also have broader implications for healing and recovery beyond the immediate postoperative period. Therefore, we posit that the adjunctive use of intravenous lidocaine goes beyond merely improving numerical outcomes. It represents a pragmatic step toward optimizing the quality of recovery and implementing a successful ERAS pathway in bariatric surgery.

## Conclusion

This study demonstrates that TAPB combined with intravenous lidocaine can provide effective postoperative analgesia and promote rapid recovery in patients undergoing bariatric surgery.

## Data Availability

The original contributions presented in the study are included in the article/supplementary material, further inquiries can be directed to the corresponding author.
